# Depression Complicates the Relationship Between Subjective Memory Complaints and Memory in Older Adults

**DOI:** 10.1093/geroni/igaa057.3290

**Published:** 2020-12-16

**Authors:** Kristi Wisniewski, Eun Young Choi, Christopher Beam, Elizabeth Zelinski

**Affiliations:** University of Southern California, Los Angeles, California, United States

## Abstract

Subjective memory complaints (SMCs) are self-reported changes in memory performance and may indicate prospective dementia. Cognitive function and depressive symptoms are negatively correlated; however, depression’s influence on the relationship between SMCs and cognition is unclear. Data were drawn from the Long Beach Longitudinal Study, a three-panel study of community-dwelling older adults from southern California who were assessed every three years. The present study included participants ages 54-89 who completed a 20-item immediate recall task at each wave and the Memory Functioning Questionnaire Frequency of Forgetting (FOF) subscale, a measure of SMC, on 2+ occasions from 1994-2014 (n=788; Mage=73 years at baseline, Meduc=15 years). Higher FOF scores indicated fewer memory complaints. Depression was measured as the average Geriatric Depression Scale score across occasions. Bivariate linear latent growth models over age adjusted for age, sex, and education (covariates associated with cognition). The SMC and recall slopes were positively correlated (parameter estimate=1.40; p<0.05). After controlling for depression, the slope-slope correlation was not significant (parameter estimate=0.83; p=0.127). However, after removing cognitively-relevant items from the Geriatric Depression Scale then adjusting for depression, the SMC slope-recall slope correlation trended toward significance (parameter estimate=0.99; p=0.070) suggesting that the cognitively relevant items were confounding the slope-slope correlation. These findings indicate that depression impacts the relationship between SMC and objective memory over age; therefore, depression may limit the clinical utility of SMCs as a cognitive screening measure. Future studies should assess depression using scales devoid of cognitive items to differentiate its influence on subjective and objective memory performance.

